# ConfRetro: a 3D-aware template-free method for enhancing retrosynthesis via molecular conformer information

**DOI:** 10.1093/bioinformatics/btag575

**Published:** 2026-08-05

**Authors:** Jiaxi Zhuang, Yu Zhang, Ying Qian, Aimin Zhou

**Affiliations:** Shanghai Frontiers Science Center of Molecule Intelligent Syntheses, Shanghai 200062, China; School of Computer Science and Technology, East China Normal University, Shanghai 200062, China; Shanghai Frontiers Science Center of Molecule Intelligent Syntheses, Shanghai 200062, China; School of Computer Science and Technology, East China Normal University, Shanghai 200062, China; Shanghai Frontiers Science Center of Molecule Intelligent Syntheses, Shanghai 200062, China; Shanghai Institute of Artificial Intelligence for Education, East China Normal University, Shanghai 200062, China; Lab of AI for Education, East China Normal University, Shanghai 200062, China; Shanghai Frontiers Science Center of Molecule Intelligent Syntheses, Shanghai 200062, China; Shanghai Institute of Artificial Intelligence for Education, East China Normal University, Shanghai 200062, China; Lab of AI for Education, East China Normal University, Shanghai 200062, China

## Abstract

**Motivation:**

Retrosynthesis plays a crucial role in organic synthesis and drug discovery, focusing on identifying a set of reactants capable of synthesizing a target product molecule. Although the existing approaches have shown promising results, they do not fully exploit 3D conformer information and molecular spatial structure, which can hinder stereochemically consistent and chemically plausible predictions.

**Results:**

To tackle this problem, we propose ConfRetro, a Transformer-based template-free method that integrates molecular conformer information and spatial structure. We devise an Atom-align Fusion module to combine 3D positional information at the model input stage, ensuring alignment between atom tokens and corresponding 3D representations. Furthermore, we design a Distance-weighted Attention mechanism to guide self-attention, constraining the receptive field of model and emphasizing chemically relevant atom pairs in 3D space. Experiments conducted on the USPTO-50K and USPTO-FULL datasets demonstrate that ConfRetro significantly outperforms existing template-free approaches, achieving a new state-of-the-art performance. Case studies further highlight its capability to predict accurate and chemically plausible reactants, even for target molecules with intricate structures. Moreover, when plugged into a standard retrosynthetic search, ConfRetro recovers feasible synthetic routes for multiple representative drug molecules (e.g. Camptothecin).

**Availability and implementation:**

ConfRetro is available at https://github.com/Jesse-zjx/ConfRetro. Archival snapshot is available at https://doi.org/10.5281/zenodo.20785018.

## 1 Introduction

Retrosynthesis, initially formulated by Corey (1991), stands as a fundamental problem within organic synthesis, aiming to discover and predict reactants given product molecules. This task presents a large chemical search space and myriad molecular combinations, posing significant challenges. Traditionally, researchers relied heavily on extensive knowledge of organic chemistry and reaction mechanisms, often resulting in inefficiency and heavy dependency on expertise. In recent years, with the significant impact of data-driven deep learning methods across various fields, computer-aided retrosynthesis has gained increasing attention, especially the template-free methods (Zhong *et al.* 2024).

Existing methods can be broadly categorized into three types based on their reliance on template priors: template-based, semi-template, and template-free. Template-based methods (Coley *et al.* 2017, Dai *et al.* 2019, Chen and Jung 2021) view retrosynthesis as a template retrieval task and subsequently employ the retrieved templates (specific chemical changes) to transform the input product molecule into reactants using cheminformatics tools like RDKit (Landrum 2013). While these methods offer strong interpretability, their reliance on predefined templates limits their coverage and scalability, rendering them insufficient for real-world scenarios. Semi-template methods (Shi *et al.* 2020, Yan *et al.* 2020) follow a two-stage pipeline: (i) reaction center identification, where product-reactant-aligned atom mapping is used to predict disconnection bonds and convert products into incomplete molecules (synthons), and (ii) synthon completion, where generative models are employed to recover full reactants from synthons. Representative works include RetroXpert (Yan *et al.* 2020), which employs an Edge-enhanced Graph Attention Network (EGAT) for bond disconnection followed by a seq2seq model for synthon completion, and G2Gs (Shi *et al.* 2020), which completes synthons in graph format. Although semi-template methods remove the strict dependency on predefined templates, the two-stage optimization process can cause suboptimal performance and weak generalization. Template-free methods (Tetko *et al.* 2020, Zheng *et al.* 2020, Mao *et al.* 2021) treat retrosynthesis prediction as a sequence-to-sequence machine translation task, typically using SMILES (Weininger 1988) to represent molecules. Transformer-based approaches (Tetko *et al.* 2020, Wan *et al.* 2022) have further improved scalability and implicit learning of chemical rules, while data augmentation and permutation invariance strategies (Seo *et al.* 2021, Tu and Coley 2022) enhance robustness. Nevertheless, most existing approaches primarily rely on 1D SMILES or 2D graphs, and only weakly encode explicit 3D geometric cues. While such representations have been effective on large reaction corpora, the lack of 3D-aware inductive biases can make it harder to model stereochemistry-sensitive transformations (e.g. chirality and spatial constraints) and may reduce robustness on structurally complex targets (Dang *et al.* 2018, Meng *et al.* 2023). In particular, models trained purely from 1D/2D inputs may produce reactant predictions with stereochemical inconsistencies or implausible configurations for molecules with rich 3D structure, such as polychiral or heteroaromatic compounds (Imrie *et al.* 2020).

*Problem*. In previous studies, the typical representation of molecules involves 1D sequences (i.e. SMILES) and 2D graphs, where explicit 3D conformational cues are largely absent. This omission may hinder accurate perception of stereochemistry and reaction feasibility. Incorporating 3D conformers into retrosynthesis prediction poses two challenges: (i) integrating 3D features while maintaining semantic alignment between atom tokens and corresponding 3D representations; and (ii) constraining the receptive field of attention mechanisms to better reflect spatial molecular structure.

In this work, we propose ConfRetro, a novel end-to-end, template-free retrosynthesis model that integrates 3D conformer information into a Transformer framework. We design two key modules: *Atom-align Fusion*, which integrates 1D and 3D representations while preserving atom-level alignment, and *Distance-weighted Attention*, which redistributes attention weights based on inter-atomic spatial distances. Moreover, we employ data augmentation strategies (Tetko *et al.* 2020, Zhong *et al.* 2022) such as SMILES permutation and atom alignment, as well as attention guidance loss (Wan *et al.* 2022) to further enhance robustness and training efficiency.

Our contributions can be summarized as follows:

- We propose ConfRetro, an end-to-end template-free retrosynthesis model that incorporates 3D conformer information to improve stereochemistry-aware prediction and robustness on structurally complex molecules.
- We introduce two novel modules: (i) *Atom-align Fusion* to integrate SMILES and 3D representations while preserving their semantic alignment; (ii) *Distance-weighted Attention* to constrain receptive fields based on 3D distances between atoms.
- Extensive experiments on USPTO-50K (Schneider *et al.* 2016) and USPTO-FULL datasets demonstrate that ConfRetro achieves state-of-the-art performance among template-free methods, under both reaction class and without reaction class settings, and is also competitive with template-based and semi-template-based approaches.

## 2 Preliminaries

### 2.1 Template-free retrosynthesis

Molecules can be represented as sequences of tokens in SMILES format. Following the SMILES tokenization of Schwaller *et al.* (2019) that separates each atom (i.e. O, C, N, Cl) and non-atom tokens (i.e. -, =, #), we let $S=[s_{1},s_{2},\ldots s_{n}]$ denote the SMILES sequence with *n* tokens. In template-free retrosynthesis, given the product SMILES *S_P_*, the goal is to model the conditional distribution of the reactant SMILES *S_R_* with an auto-regressive sequence-to-sequence model:


(1) $$p_{\theta }(S_{R}|S_{P})=\prod_{t=1}^{\left|S_{R}\right|}p_{\theta }(r_{t}|r_{<t},S_{P}),$$

where *r_t_* denotes the *t*-th token in *S_R_* and $r_{<t}$ are the previously generated reactant tokens.

### 2.2 Molecular conformer and ComENet

A molecule is a dynamic structure since atoms are in continual motion in 3D space. The local minima on the potential energy surface are called conformers. Generally, a molecular conformer can be represented as $G_{3D}=(A,C)$, where $A={\left\{a_{i}\right\}}_{i=1,\ldots ,n}$ is the atoms list, $C={\left\{(x_{i},y_{i},z_{i})\right\}}_{i=1,\ldots ,n}$ is the 3D coordinates list. To fulfill the global completeness of 3D conformer information of a molecule, ComENet (Wang *et al.* 2022) encodes rotation angles and spherical coordinates within the 1-hop neighborhood of atoms via the message passing scheme (Balcilar *et al.* 2021).

## 3 Method

**Figure 1:**
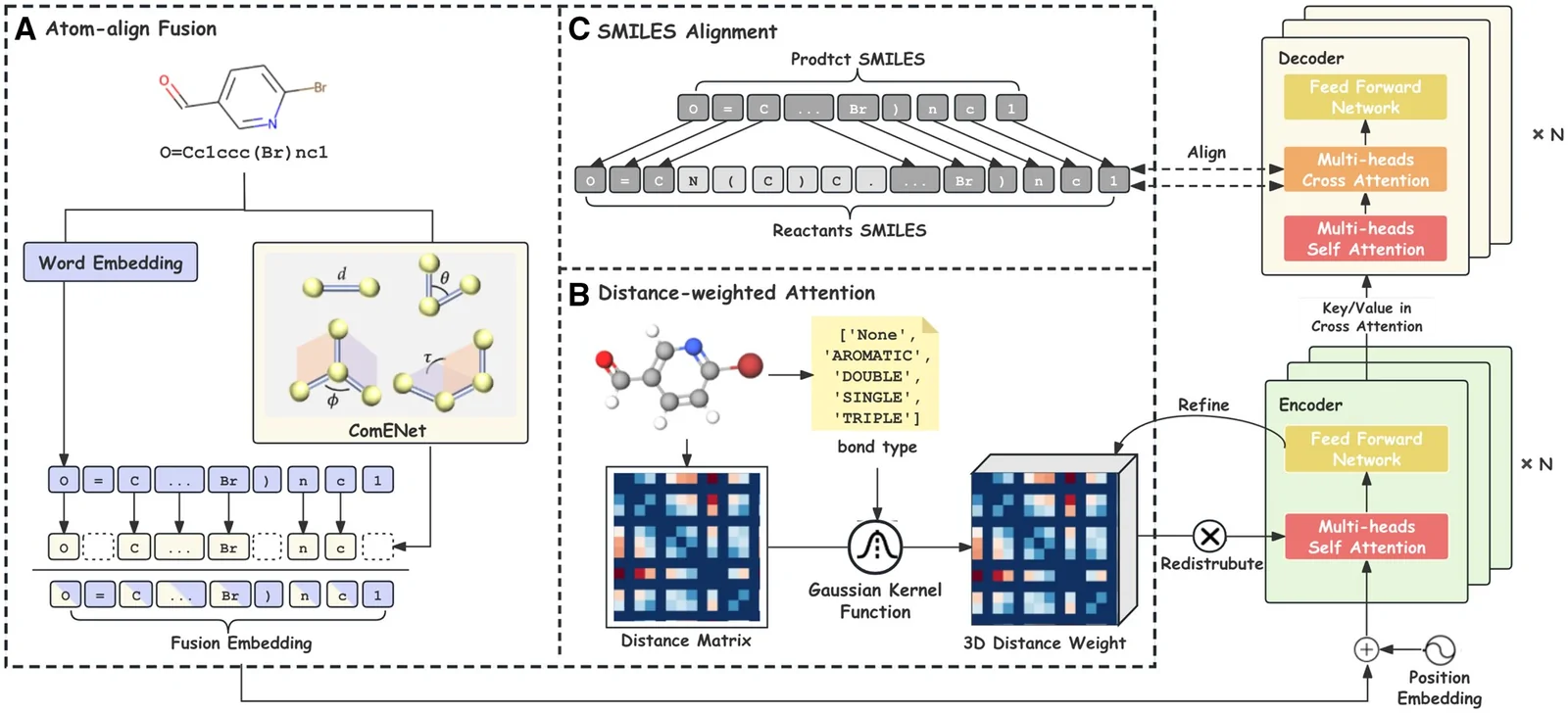
Architecture overview of ConfRetro based on Transformer. (A**)** SMILES and the molecular conformer are processed through Word Embedding and ComENet to yield SMILES sequence embedding and 3D position embedding. Following the Atom-align integration, the fusion embedding is then input into the Encoder. (B) In the Encoder, 3D Distance weight is obtained for redistribution of self-attention by the Gaussian basis kernel function, while being refined layer by layer. (C) In the Decoder, SMILES Alignment is employed as guidance to align the cross-attention between the product and reactants.

In this section, we introduce ConfRetro, a novel transformer-based model that is capable of learning richer molecular representation through SMILES sequence and spatial conformers. An overview of the proposed method is shown in Fig. 1.

Figure 1a illustrates Atom-align Fusion, which incorporates both 1D SMILES token embedding and 3D position embedding to obtain a fusion embedding as input to the encoder-decoder model. By adopting this approach, the model is capable of integrating 3D positional information while preserving alignment (Section 3.1). Figure 1b illustrates Distance-weighted Attention, which directs self-attention to prioritize chemically relevant atoms within spatial structure (Section 3.2). Figure 1c illustrates SMILES Alignment strategy, which further bridges the mapping between product and reactant SMILES in the cross-attention module of decoder (Section 3.3). The overall training and inference process is conducted in an end-to-end manner, and it is a template-free method that does not rely on RDKit for molecular editing.

### 3.1 Atom-align fusion

The most direct method of feature integration is concatenation (Mao *et al.* 2021), but this approach disrupts the alignment between 1D representations of atom tokens and their corresponding 3D representations. The challenge lies in how to integrate conformer information into the molecular representation while preserving alignment between them.

To address this issue, we introduced the Atom-align Fusion module. The purpose is to obtain the fusion embedding $F_{3D}\in R^{M\times D}$ by associating SMILES token embedding $T\in R^{M\times D}$ with 3D position embedding $P_{3D}\in R^{N\times D}$, where *D* the is model dimension, *M* is the number of SMILES tokens, and *N* is number of atoms.

Firstly, to obtain the complete 3D molecular representation, we extracted 3D position embedding $P_{3D}$ from the molecular conformer using ComENet (Wang *et al.* 2022). ComENet proposes a novel message passing scheme (Balcilar *et al.* 2021) that operates within a 1-hop neighborhood with radial distance *d*, polar angle *θ*, azimuthal angle ϕ and rotation angle *τ*, and then builds message passing scheme as follows:


(2) $$v_{i}^{t\;1}=g\left(v_{i}^{t},\sum_{j\in N_{i}}f\left(v_{j},d_{ij},\theta_{ij},\phi_{ij},\tau_{ij}\right)\right),$$

where $v_{i}^{t}\to v_{i}^{t\;1}$ denotes the update of atom (node) representation, *g* and *f* are implemented by neural networks or mathematical operations, $d_{ij},\;\theta_{ij}$ and $\phi_{ij}$ specify the 1-hop local neighborhood, and $\tau_{ij}$ determines its orientation. We omit the final aggregation layer of ComENet to obtain the 3D representation corresponding to each atom $P_{3D}={\left\{v_{i}\right\}}_{i=1,2,\ldots ,N}$ rather than the entire molecular graph.

Subsequently, we input $P_{3D}$ along with the SMILES token embedding *T* into the encoder. To facilitate a more comprehensive integration of SMILES and conformer, we align the embeddings *T* and $P_{3D}$ by atom index in the same vector space. We first enlarge the length of $P_{3D}$ from *N* (the number of atoms) to *M* (the number of tokens) by padding zeros at the indices of non-atom tokens. At the model input stage, we blend $P_{3D}$ and *T* to obtain the fusion embedding $F_{3D}$, allowing effective integration of 3D positional and sequential information at each atom position while preserving the alignment between them:


(3) $$F_{3D}=\lambda_{1}\times \mathrm{pad}(P_{3D})\;\lambda_{2}\times T,$$

where *λ*_1_ and *λ*_2_ are trainable parameters that adaptively adjust the weights of 3D position embedding $P_{3D}$ and SMILES token embedding *T* during training. In addition, we still use Sinusoidal Position Embedding (Vaswani *et al.* 2017) to encode the positions of tokens.

### 3.2 Distance-weighted attention

According to Li *et al.* (2017) and Wan *et al.* (2022), multi-head attention calculation in the vanilla Transformer is insufficient and lacks chemical knowledge. Simultaneously, 3D distances between atoms within a molecule reflect the spatial relation between any pair of atoms in physical chemistry, such as bond energy, stability, electron affinity, and interatomic force. Incorporating 3D distances into the multi-head attention calculation process enables the model to reschedule attention accordingly, which not only alleviates the redundancy in multi-head attention but also enables distance-based attention redistribution during the attention calculation.

Similar to the method of partitioning attention heads in Wan *et al.* (2022), we divide the multi-head attention into normal attention heads and spatial attention heads. Normal attention heads follow the traditional transformer manner to learn sequence-wide information, whereas spatial attention heads employ 3D distances as an external attention weight to enable attention to be redistributed based on distances. The whole module can be formulated as two steps: (1) Construct 3D Distance Weight, (2) Attention Redistribute & Weight Refine.

#### 3.2.1 Construct 3D distance weight

First, we construct a distance matrix $D\in R^{N\times N}$ from molecular conformer as:


(4) $$D={\left\{d_{i,j}\right\}}_{i,j=1,2,\ldots ,N}\;d_{ij}=||c_{i}-c_{j}|{|}_{2},$$

where *N* is the number of atoms, *i*, *j* are the indices of an atom pair, and $c_{i}$ is the coordinate of the *i*-th atom. To explore high-dimensional space, we employ Gaussian basis function (Scholkopf *et al.* 1997) to transform the distance $d_{ij}$ between each atom pair (*i*, *j*) into a higher dimension as:


(5) $$\psi_{\left(i,j\right)}^{k}=\frac{1}{\sqrt{2\pi }\left|\sigma^{k}\right|}\text{exp}\;\left(-\frac{1}{2}{\left(\frac{\gamma_{\left(i,j\right)}d_{ij}\;\beta_{\left(i,j\right)}-\mu^{k}}{\left|\sigma^{k}\right|}\right)}^{2}\right),$$

where *k*= {1, _**…**_, *K*} and *K* is the number of Gaussian kernels. *μ^k^* and *σ^k^* represent the center and scaling factor of the *k*-th Gaussian basis kernel. $\gamma_{\left(i,j\right)}$ and $\beta_{\left(i,j\right)}$ are learnable scalars related to bond type (e.g. SINGLE, DOUBLE) of atom pair (*i*, *j*). To obtain the 3D Distance weight $\Phi_{\left(i,j\right)}$, we use two layers of Non-Linear Layer with the activation function Gaussian Error Linear Units (GELU) (Hendrycks and Gimpel 2016) to encode $\psi_{\left(i,j\right)}$ as:


(6) $$\Phi_{\left(i,j\right)}=W_{2}(\mathrm{GELU}(W_{1}(\psi_{\left(i,j\right)}))),$$

where $\psi_{\left(i,j\right)}=\mathrm{stack}([\psi_{\left(i,j\right)}^{1};\ldots ;\psi_{\left(i,j\right)}^{k}]),\;W_{1},W_{2}\in R^{K\times K}$ are learnable parameters, and 3D Distance weight $\Phi_{\left(i,j\right)}\in R^{N\times N\times K}$.

#### 3.2.2 Attention redistribute and weight refine

Second, we leverage the previously obtained 3D Distance weight $\Phi_{\left(i,j\right)}$ to guide the model in redistributing attention weights by the internal spatial structure, instead of directly calculating attention scores between each token, like vanilla Transformer. We divide the attention heads into two halves: normal attention head and spatial attention heads. The normal attention head still capture the context of the SMILES sequence like original attention computation. The spatial attention heads, on the other hand, focus more on the molecular internal structure, limiting the receptive field of each atom and considering the 3D distance with other atoms. For the *i*-th token of the *l*-th encoder layer, the roll-out form of spatial attention head is:


(7) $$\begin{array}{c} x_{i,\text{spatial}}^{l\;1}=\sum_{j\in N(i)}\mathrm{softmax}\left(\Phi_{\left(i,j\right)}^{l}⊙\frac{q_{i}k_{j}^{T}}{\sqrt{d}}\right)v_{j},\\ \left[q_{i},k_{j},v_{j}\right]=\left[h_{i}^{l}W^{Q},h_{j}^{l}W^{K},h_{j}^{l}W^{V}\right], \end{array}$$

where $\Phi_{\left(i,j\right)}^{l}$ is almost the same as the 3D Distance weight $\Phi_{\left(i,j\right)}$ but padding 0 for non-atom token for element-wise multiplication $⊙,\;W^{Q},\;W^{K}$ and $W^{V}$ are projection matrices for query *q*, key *k*, value *v* in self-attention. Computed representations of normal attention head and spatial attention heads are concatenated along the hidden dimension and then processed through a linear layer, forming the updated context for the next layer $h^{l\;1}$, formally we have:


(8) $$\begin{array}{c} h^{l\;1}=W_{\mathrm{Linear}}\left(\left[x_{\text{normal}}^{l\;1};x_{\text{spatial}}^{l\;1}\right]\right). \end{array}$$

In addition, we perform Weight Refine to update our 3D Distance weight layer by layer. The Weight Refine module is a fully connected (FC) layer that takes concatenation of updated contexts of atom pair (*i*, *j*) to refine the 3D attention weight within the receptive field, which is formulated as:


(9) $$\Phi_{\left(i,j\right)}^{l\;1}=\Phi_{\left(i,j\right)}^{l}\;\mathrm{FC}\left(\left[h_{i}^{l\;1};h_{j}^{l\;1}\right]\right).$$

### 3.3 SMILES alignment

During the occurrence of chemical reactions, the majority of atoms remain unchanged (Chen and Jung 2021, Zhong *et al.* 2022). We can utilize the atom-map to annotate the correspondence between atoms of the product and reactants in the reaction, which remains unchanged during the reaction. Furthermore, the correspondence of atoms can be readily transferred to the token correspondence in the SMILES sequence (see Fig. 1c) and construct SMILES Alignment Map (SAM) as:


(10) $$SAM_{ij}=\{\begin{array}{cc} 1 & \;\text{if}\;R_{i}\underset{\;\text{mapped}\;}{↔}P_{j}\\ 0 & \;\text{else}\; \end{array}$$

Inspired by Deshpande and Narasimhan (2020), we similarly employed the guided attention approach by introducing guidance loss $L_{SA}$ to encourage alignment between the cross attention scores in the final layer of the decoder and SMILES Alignment Map, strengthening the attention weights between corresponding tokens.

### 3.4 Overall loss

The overall loss is composed of the language modeling R-Drop loss (Wu *et al.* 2021) $L_{LM}$ for retrosynthesis, along with the SMILES Alignment guidance loss $L_{SA}$.

The objectives of overall loss are: (i) approximate the target in retrosynthesis, (ii) ensure robustness under different dropout, (iii) strengthen the alignment relationship in cross-attention. The formula is as follows:


(11) $$L=L_{LM}^{\left(CE\right)}\;\alpha L_{LM}^{\left(KL\right)}\;\beta L_{SA},$$

where $L_{LM}^{\left(CE\right)}$ is Cross-Entropy loss to minimize prediction error against actual outputs, $L_{LM}^{\left(KL\right)}$ is KL-divergence in R-Drop loss to strive for outputs of models with varying Dropout to be as similar as possible. $L_{SA}$ is Cross-Entropy loss between cross-attention score of last decoder layer and SAM that we mention in Section 3.3. We set *α* to 0.5, *β* to 1.0.

## 4 Experiments

### 4.1 Datasets

We evaluate our model on the widely used retrosynthesis benchmark dataset USPTO-50K (USPTO-50K and USPTO-FULL datasets can be obtained at https://github.com/Hanjun-Dai/GLN) (Schneider *et al.* 2016), which contains 50 016 atom-mapped reactions. Following (Coley *et al.* 2017), the data are split into 40 008, 5001, and 5007 reactions for the training, validation, and test sets, respectively. Molecular conformers are generated using RDKit as follows: each SMILES is parsed with MolFromSmiles, hydrogen atoms are added via AddHs, a single 3D conformer is embedded with AllChem.EmbedMolecule (random seed fixed at 10; up to 10 retries on failure), followed by MMFF94 force-field optimization with AllChem.MMFFOptimizeMolecule (RDKit default, maxIters = 200), and finally hydrogen atoms are removed with RemoveHs. The same deterministic conformer (seed = 10) is used consistently at both training and testing time. Stereochemistry specified in the input SMILES (@/@@, E/Z) is enforced during conformer generation via MolFromSmiles; for atoms with unspecified stereochemistry, RDKit assigns an arbitrary but deterministic 3D configuration under the fixed random seed. The robustness of performance to this single-conformer choice is examined in Supplementary Material I2, available as supplementary data at *Bioinformatics* online, and a stereochemistry-stratified evaluation is reported in Supplementary Material I3, available as supplementary data at *Bioinformatics* online. The ground-truth SMILES Alignment Map is extracted using the algorithm described in Wan *et al.* (2022). In addition, we conduct experiments on the larger USPTO-FULL dataset (Dai *et al.* 2019), which comprises 10 13 118 atom-mapped reactions without reaction-class information. This more extensive collection not only enables a comprehensive evaluation of the proposed method’s performance, generalization potential, and scalability but also provides samples with more intricate molecular structures, allowing further validation of our model’s capability to handle complex spatial configurations.

### 4.2 Settings

Our model is built on the vanilla Transformer, consisting of 6 encoder layers and 6 decoder layers with 8 attention heads. The model dimension is set to 512. We employ the Adam optimizer (Kingma and Ba 2014) with $\left(\beta_{1},\beta_{2})=(0.9,0.98\right)$ and train the model with a batch size of 16. During training, we use an early stopping of 7 epochs under a maximum of 1000 epochs to obtain the 7 best models and average their parameters. The choice of averaging 7 checkpoints is empirical as shown in the Supplementary Material I4, available as supplementary data at *Bioinformatics* online. Finally, the whole training process took approximately 20 hours on a single NVIDIA GeForce RTX 4090 GPU for USPTO-50K; training on USPTO-FULL (approximately 20_**×**_ larger) takes approximately one week on the same hardware. More details can be found in Supplementary Material E, available as supplementary data at *Bioinformatics* online.

### 4.3 Evaluation

During inference, we use beam search (Tillmann and Ney 2003) decoding strategy with a beam size of 10. After that, we evaluate our model with *Top-k Accuracy*, which considers it correct only if Top-k predicted reactants from the beam search results are the same as those in the original test set. We verify *Top-k Validity* based on whether RDKit can recognize the Top-k predicted reactants. *Top-k Round-Trip Accuracy* measures the percentage of predicted reactants that can lead back to the original product.

### 4.4 Baseline

We compare our proposed method, ConfRetro, with several strong baseline models from three types of retrosynthesis methods. We take RetroSim (Coley *et al.* 2017), GLN (Dai *et al.* 2019) and LocalRetro (Chen and Jung 2021) to represent template-based methods. We take RetroXpert (Yan *et al.* 2020), G2Gs (Shi *et al.* 2020), GraphRetro (Somnath *et al.* 2020), RetroPrime (Wang *et al.* 2021), MEGAN (Sacha *et al.* 2021), MARS (Liu *et al.* 2024), and Graph2Edits (Zhong *et al.* 2023) to represent semi-template methods. We take SCROP (Zheng *et al.* 2020), Aug.Transformer (Tetko *et al.* 2020), Graph2SMILES (Tu and Coley 2022), GTA (Seo *et al.* 2021), Retroformer (Wan *et al.* 2022), and NAG2G (Yao *et al.* 2024) to represent template-free methods. We did not choose pre-trained methods for comparison as they are trained on larger datasets. We apply Root-align data augmentation (Zhong *et al.* 2022) and conduct experiments on the proposed model ConfRetro, choosing on-the-fly augmentation instead of directly expanding the training dataset; following the data augmentation strategy of previous works, we additionally apply 20**×** augmentation on the test set of USPTO-50K and 5**×** augmentation on the test set of USPTO-FULL.

### 4.5 Performance

#### 4.5.1 Performance on USPTO-50K

##### 4.5.1.1 *Top-k accuracy*

As shown in Table 1, when the reaction class is known, ConfRetro achieves Top1-10 results that outperform all template-free/semi-template baselines and most state-of-the-art template-based baselines. Notably, our model exceeds both template-based and semi-template methods, despite their reliance on predefined templates or RDKit editing. When the reaction class is unknown, our model still achieves improvements in Top1-3 accuracy within the template-free category. Achieving high-confidence predictions (i.e. Top-1 accuracy), which narrows the search space for synthesis routes and enhances efficiency, makes our model particularly well-suited for real-world scenarios. Performance across each reaction class in the USPTO-50K dataset is provided in Supplementary Material B1, available as supplementary data at *Bioinformatics* online.

**Table 1:** Top-k accuracy for retrosynthesis prediction on USPTO-50K.^a^

Model	**Top-k accuracy (%)**							
	Reaction class known				Reaction class unknown			
	1	3	5	10	1	3	5	10
Template-based								
RetroSim (Coley *et al.* 2017)	52.9	73.8	81.2	88.1	37.3	54.7	63.3	74.1
GLN (Dai *et al.* 2019)	**64.2**	79.1	85.2	90.0	52.5	69.0	75.6	83.7
LocalRetro (Chen and Jung 2021)	63.9	**86.8**	**92.4**	**96.3**	**53.4**	**77.5**	**85.9**	**92.4**
Semi-template-based								
RetroXpert (Yan *et al.* 2020)	62.1	75.8	78.5	80.9	50.4	61.1	62.3	63.4
G2G (Shi *et al.* 2020)	61.0	81.3	86.0	88.7	48.9	67.6	72.5	75.5
GraphRetro (Somnath *et al.* 2020)	63.9	81.5	85.2	88.1	53.7	68.3	72.2	75.5
RetroPrime (Wang *et al.* 2021)	64.8	81.6	85.0	86.9	51.4	70.8	74.0	76.1
MEGAN (Sacha *et al.* 2021)	60.7	82.0	87.5	91.6	48.1	70.7	78.4	86.1
MARS (Liu *et al.* 2024)	66.2	85.8	90.2	92.9	54.6	76.4	83.3	88.5
Graph2Edits (Zhong *et al.* 2023)	**67.1**	**87.5**	**91.5**	**93.6**	**55.1**	**77.3**	**83.4**	**89.4**
Template-free								
SCROP (Zheng *et al.* 2020)	59.0	74.8	78.1	81.1	43.7	60.0	65.2	68.7
Aug.Transformer (Tetko *et al.* 2020)	–	–	–	–	48.3	–	73.4	77.4
GTA (Seo *et al.* 2021)	–	–	–	–	51.1	67.6	74.8	81.6
Graph2SMILES (Tu and Coley 2022)	–	–	–	–	52.9	66.5	70.0	72.9
Retroformer (Wan *et al.* 2022)	64.0	82.5	86.7	90.2	53.2	71.1	76.6	82.1
NAG2G (Yao *et al.* 2024)	67.2	86.4	90.5	93.8	55.1	76.9	83.4	89.9
ConfRetro (Ours)	**68.2**	**88.9**	**92.8**	**96.1**	**56.2 ± 0.2** ^b^	**79.3 ± 0.4**	**86.2 ± 0.4**	**91.7 ± 0.2**

a Results for all baseline methods are taken from the respective original publications; ConfRetro results are from this work. Best performance is in bold.

b Bootstrap 95% CI for Top-1 (reaction class unknown): [54.8, 57.5] (percentile bootstrap, *B *= 10 000 over test reactions; see Supplementary Material I5, available as supplementary data at *Bioinformatics* online for full results).

##### 4.5.1.2 *Top-k validity*

We take four typical template-free methods, Graph2SMILES, RetroPrime, Retroformer, and NAG2G, as baseline models to verify the predicted SMILES validity of our model. We refrained from selecting template-based and semi-template methods, as SMILES constructed based on template information are guaranteed to be valid. In contrast, template-free methods often struggle to generate valid SMILES without extra chemical guidance. As shown in Table 2, our model is more capable of generating valid SMILES than the baseline models, especially for Top-5 and Top-10 reactants, where it is far ahead of the others. This indicates that incorporating 3D conformer information can assist the model in grasping the structures involved in chemical reactions, thus preventing the generation of SMILES that violate chemical principles.

**Table 2:** Top-k SMILES validity/round-trip accuracy for retrosynthesis prediction on USPTO-50K with reaction class unknown.^a^

Model	Top-k validity (%)				Top-k round-trip (%)			
	1	3	5	10	1	3	5	10
Graph2SMILES	99.4	90.9	84.9	74.9	76.7	56.0	46.4	34.9
RetroPrime	98.9	98.2	97.1	92.5	79.6	59.6	50.3	40.4
Retroformer	99.2	98.5	97.4	96.7	78.9	72.0	67.1	57.2
NAG2G	99.7	98.6	97.1	92.9	–	–	–	–
ConfRetro (ours)	**99.8**	**99.6**	**99.1**	**97.0**	**90.7**	**81.3**	**75.7**	**69.0**

a ConfRetro results are from this work. Best performance is in bold.

##### 4.5.1.3 *Top-k round-trip accuracy*

To assess the accuracy of our predicted synthesis plans, we use the Molecule Transformer (Schwaller *et al.* 2019) as the benchmark reaction prediction model and calculate the Top-k Round-Trip Accuracy. The results in Table 2 clearly show that our model significantly outperforms other baselines, achieving a remarkable 12% improvement in Top-1 Round-Trip Accuracy compared to the best baseline. A higher Round-Trip Accuracy indicates that the predicted synthesis plans are more chemically valid and executable, as they can be successfully converted back to the target molecules by a forward reaction prediction model.

#### 4.5.2 Performance on USPTO-FULL

To further assess the scalability and generalization of our method in large-scale and structurally diverse chemical space, we evaluate ConfRetro on the USPTO-FULL dataset, which contains over 1 million atom-mapped reactions and does not provide reaction-class labels. Beyond its scale, USPTO-FULL is typically noisier than USPTO-50K, posing a more challenging setting for robust retrosynthesis modeling. Compared with USPTO-50K, USPTO-FULL exhibits substantially greater reaction diversity and more complex scaffolds, including bulky ring systems, dense stereocenters, and coupled functional-group transformations, making it a stricter stress test for retrosynthesis models.

Table 3 reports the Top-*k* accuracy of ConfRetro and representative baselines spanning template-based, semi-template, and template-free families. Overall, ConfRetro achieves state-of-the-art performance within the template-free category, reaching 51.7%, 69.2%, 74.5%, and 79.7% Top-1/3/5/10 accuracy, respectively. Notably, it surpasses NAG2G, the strongest prior template-free system, by 2.0%, 4.6%, 5.2%, and 4.3% at Top-1/3/5/10, respectively. These consistent gains across *k* suggest that ConfRetro not only improves the confidence of its top-ranked prediction but also yields a higher-quality candidate set under beam search.

**Table 3:** Top-k accuracy for retrosynthesis prediction on USPTO-FULL without reaction class.^a^

Model	Top-k accuracy (%)			
	1	3	5	10
Template-based				
RetroSim	32.8	–	–	56.1
GLN	35.8	–	–	60.8
LocalRetro	39.3	–	–	63.7
Semi-template-based				
RetroPrime	44.1	59.1	62.8	68.5
MEGAN	33.6	–	–	63.9
Graph2Edits	44.0	60.9	66.8	72.5
Template-free				
Aug.Transformer	46.2	–	–	73.3
GTA	46.6	–	–	70.4
Graph2SMILES	45.7	–	–	63.4
NAG2G	49.7	64.6	69.3	74.0
ConfRetro (ours)	**51.7**	**69.2**	**74.5**	**79.7**

a Results for all baseline methods are taken from the respective original publications. Best performance is in bold.

It is also worth emphasizing that ConfRetro remains competitive even when compared with template-based and semi-template approaches such as LocalRetro (Chen and Jung 2021) and Graph2Edits (Zhong *et al.* 2023). Unlike these methods, ConfRetro is trained end-to-end without predefined reaction templates or hard-coded edit operations. By integrating 3D conformer information and explicit product-reactant-alignment supervision, the model better accounts for spatial constraints, improving robustness on sterically congested and geometrically intricate motifs that are often ambiguous from 2D topology alone.

We attribute the performance gains on USPTO-FULL to two main factors. First, conformer-aware encodings (Atom-align Fusion and Distance-weighted Attention) increase sensitivity to spatial proximity and steric coupling, which becomes increasingly important for large-ring and polyfunctional molecules. Second, SMILES Alignment supervision stabilizes decoding for long and heterogeneous products by encouraging consistent structural correspondence between product and reactant tokens, mitigating beam-search degeneration and improving the quality of alternatives at larger *k*.

Overall, the USPTO-FULL results demonstrate that ConfRetro scales beyond curated benchmarks and remains reliable in a setting that more closely reflects real-world retrosynthesis workloads, characterized by the absence of reaction-class hints, high transformation diversity, increased noise, and structurally complex targets.

### 4.6 Ablation study

To quantify the contribution of each proposed component, we perform an ablation study on USPTO-50K under the reaction-class-unknown setting. Table 4 reports the Top-*k* accuracy and SMILES validity of different model variants denoted by **➀** to **➅**, corresponding to different combinations of modules (a) Atom-align Fusion, (b) Distance-weighted Attention, and (c) SMILES Alignment.

**Table 4:** Ablation study of ConfRetro with reaction class unknown on USPTO-50K dataset.^a^

	(a)	(b)	(c)	Top-k accuracy (%)				Top-k validity (%)			
				1	3	5	10	1	3	5	10
➀	–	–	–	50.9	72.7	78.8	82.7	99.1	98.4	97.7	95.6
➁	–	–	*✓*	53.5	75.9	83.5	89.0	99.3	98.9	98.5	95.3
➂	*✓*	–	*✓*	54.0	75.2	81.9	87.2	99.6	99.2	98.4	94.9
➃	–	*✓*	*✓*	54.8	75.3	81.9	87.8	99.7	99.4	98.6	94.2
➄	*✓*	*✓*	–	54.8	75.1	82.8	89.1	99.7	99.6	98.9	95.9
➅	*✓*	*✓*	*✓*	**56.2**	**79.3**	**86.2**	**91.7**	**99.8**	**99.6**	**99.1**	**97.0**

a Module (a) is Atom-align fusion; Module (b) is Distance-weighted attention and Module (c) is SMILES alignment. Best performance is in bold.

Comparing **➁** with the baseline **➀**, introducing SMILES Alignment (c) leads to a consistent improvement in Top-*k* accuracy, indicating that explicit alignment supervision stabilizes decoding under beam search. Further incorporating Atom-align Fusion (a) (**➂** vs. **➁**) improves Top-1 accuracy, suggesting that conformer-informed atom-level correspondence helps the model better prioritize the most plausible reactant set. In contrast, adding Distance-weighted Attention (b) (**➃** vs. **➁**) yields gains across all Top-*k* values, with more pronounced improvements at larger *k*, reflecting enhanced modeling of internal molecular structure.

The full model **➅**, which combines all three modules, achieves the best overall performance, simultaneously improving high-confidence predictions and maintaining strong results at larger beam widths. These results demonstrate that conformer-aware encoding and alignment-based supervision are complementary and jointly contribute to the effectiveness of ConfRetro.

To further investigate *why* the conformer-aware modules help, we disentangle 3D geometry from model capacity, atom-level supervision, bond-type features, and test-time augmentation by varying a single factor at a time while holding all other components constant. Table 5 reports the results on USPTO-50K (reaction class unknown).

**Table 5:** Sources of improvement on USPTO-50K (reaction class unknown).^a^

Variant	Top-1	Top-3	Top-5	Top-10
w/o bond-type	56.0	79.2	**86.5**	**91.8**
w/o 3D distance	53.0	77.4	84.5	90.5
w/random conformer	53.4	77.7	84.8	90.7
w/o TTA ($n_{\text{aug}}$ = 1)	55.5	77.2	83.4	89.1
ConfRetro (Ours)	**56.2**	**79.3**	86.2	91.7

a Each row varies a single factor relative to ConfRetro (ours): w/o bond-type fixes the bond-type affine to identity, w/o 3D distance zeroes the distance term, w/random conformer replaces coordinates with noise, and w/o TTA disables test-time augmentation.  Best performance is in bold.

Three findings emerge. (i) *3D geometry is the causal source.* Replacing the real conformer with random coordinates drops Top-1 accuracy by 2.7 points (56.2 **→** 53.4). Since every factor other than the atomic coordinates is held fixed, this gap is attributable to genuine 3D geometry rather than added parameters or supervision, and is statistically significant (McNemar *P* = 9.0 × 10^−8^). (ii) *The benefit comes from distance, not bond type.* The distance module combines an inter-atomic distance term with a learnable bond-type affine. Removing the bond-type affine (w/o bond-type) leaves performance essentially unchanged (56.0 vs. 56.2 Top-1), whereas removing the distance term (w/o 3D distance) collapses accuracy to 53.0, comparable to the random-conformer control, so the useful signal lies in the actual 3D distances. (iii) *The gains are not an artifact of augmentation.* Even with test-time augmentation disabled ($n_{\text{aug}}$ = 1), ConfRetro still attains Top-1 accuracy of 55.5%, well above both controls. Bootstrap confidence intervals and paired significance tests for all variants are reported in Supplementary Material I5, available as supplementary data at *Bioinformatics* online.

### 4.7 Case study

To verify that the proposed method ConfRetro can handle molecules with more intricate spatial structures, we chose four types of products as representatives:

- Polychiral: a product with two or more chiral centers (“@/@@” in SMILES), leading to complex stereochemistry and multiple isomers.
- Heteroaromatic: a product containing at least one heteroatom as part of its aromatic ring structure.
- Fused/Bridged Rings: product features multiple ring structures joined together, either sharing common atoms or connected by bridge atoms.
- Complex: a product encompassing two or more features mentioned above.

**Figure 2:**
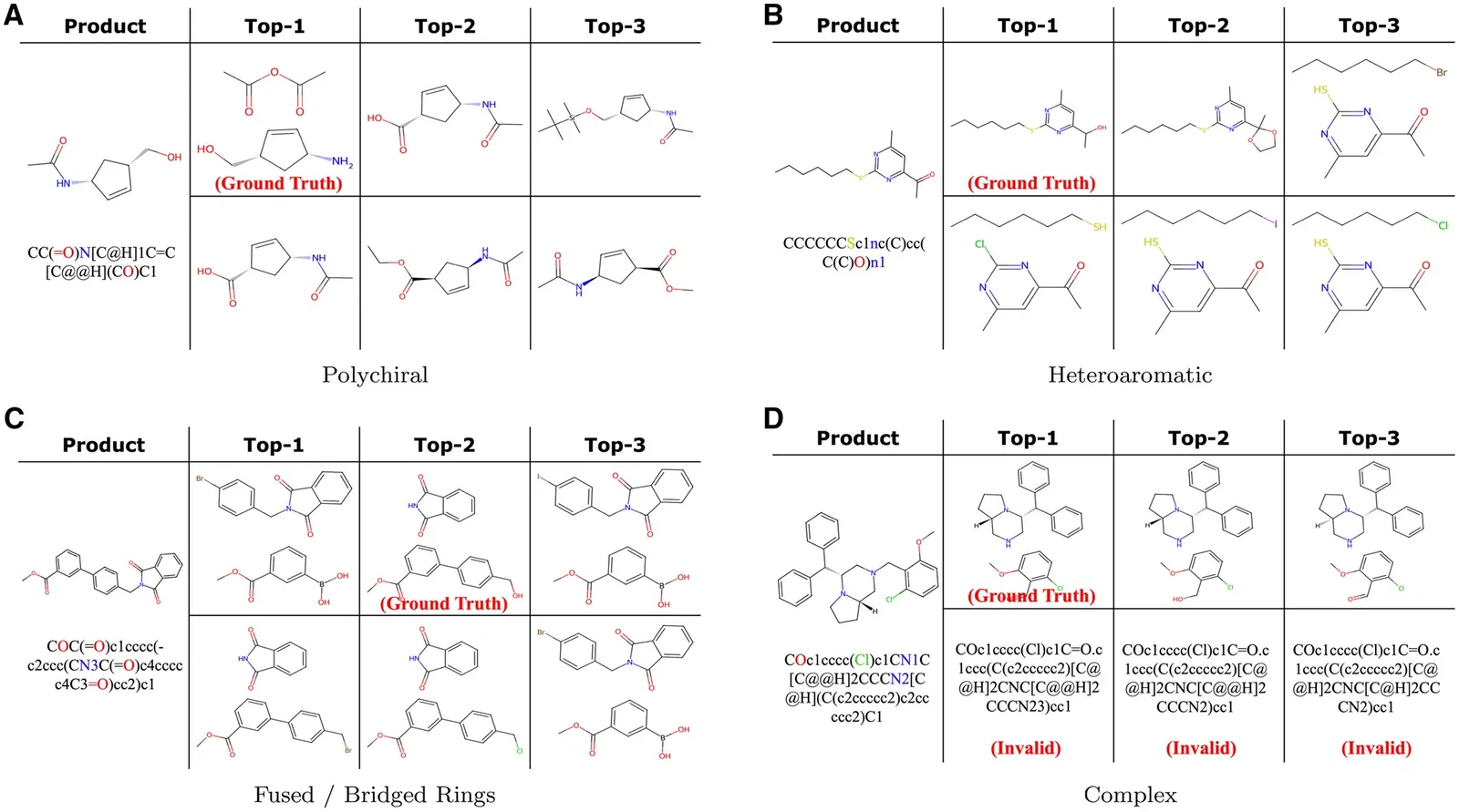
Case study for intricate structures. (A)–(D) illustrate the retrosynthesis predictions of ConfRetro (top) and Transformer (bottom) on four representative product molecules with complex ring systems or stereocenters.

We conducted experiments comparing ConfRetro with a Vanilla Transformer, analyzing Top-1 to Top-3 predictions, as illustrated in Fig. 2. The rows in each subfigure represent the results of ConfRetro and Transformer, respectively, with ground truth and invalid SMILES highlighted in bold red.

The results show that when dealing with molecules of intricate spatial structures, ConfRetro can infer reasonable reactants with high confidence (often at Top-1 or Top-2), while the Transformer without 3D information struggles to generate accurate predictions. Notably, for Complex products, ConfRetro successfully deduces the molecular skeleton and achieves the ground truth at Top-1, while the Vanilla Transformer fails to produce any valid SMILES within the Top-3 predictions.

**Figure 3:**
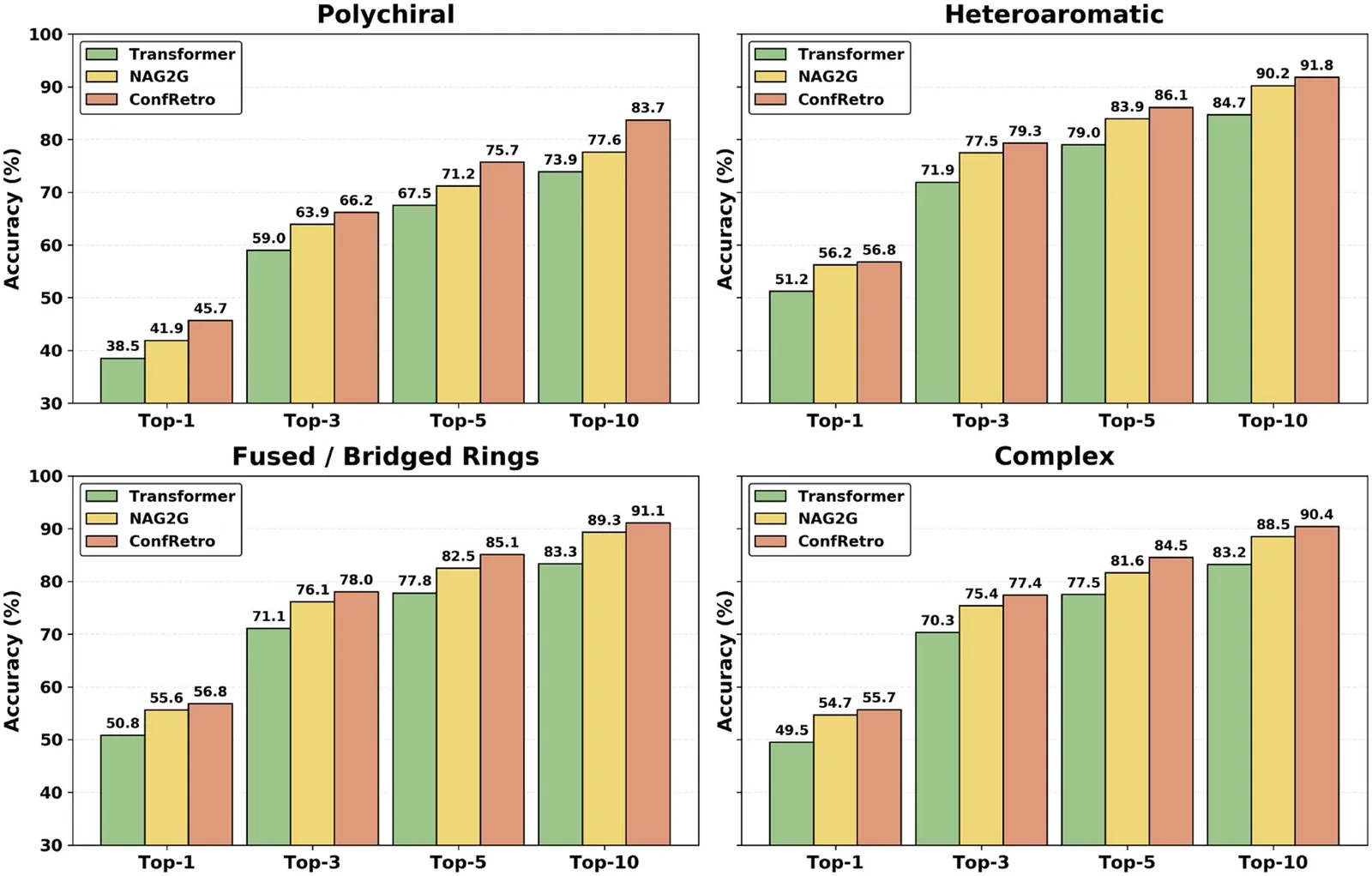
Quantitative comparison of ConfRetro, NAG2G, and Vanilla Transformer on four types of complex molecules.

To further demonstrate the effectiveness of ConfRetro, we conducted a quantitative comparison across all four categories against both the Vanilla Transformer and NAG2G, another 3D-aware template-free method. As shown in Fig. 3, ConfRetro consistently outperforms both baselines across all categories and all Top-*k* values. Importantly, both 3D-aware methods (ConfRetro and NAG2G) outperform the 2D Transformer, yet ConfRetro surpasses NAG2G in all four categories, indicating that the performance gain stems from *how* 3D geometry is used (specifically, the Atom-align Fusion and Distance-weighted Attention designs) rather than simply having 3D inputs available. The performance gap is most pronounced for Polychiral molecules (the hardest category), where incorporating 3D spatial information is crucial for resolving stereochemical ambiguities. Detailed per-category Top-*k* accuracy and SMILES validity for the three methods are reported in Supplementary Material G, available as supplementary data at *Bioinformatics* online.

## 5 Synthesis routes

To demonstrate the practical applicability and robustness of our framework in planning feasible multi-step synthetic routes for structurally intricate, real-world pharmaceuticals, we conducted a case study on a set of complex target molecules. A key objective of this experiment was to validate that our method can successfully navigate the challenging chemical search space of polycyclic natural products with stringent stereochemical requirements, thereby generating complete and chemically valid retrosynthetic pathways.

For this purpose, we selected Camptothecin as a representative and highly challenging test case. Camptothecin is a clinically significant anticancer natural product that functions as a potent DNA topoisomerase I inhibitor (Li *et al.* 2016). Its complex structure and intricate biosynthesis make it a challenging molecule for retrosynthesis.

**Figure 4:**
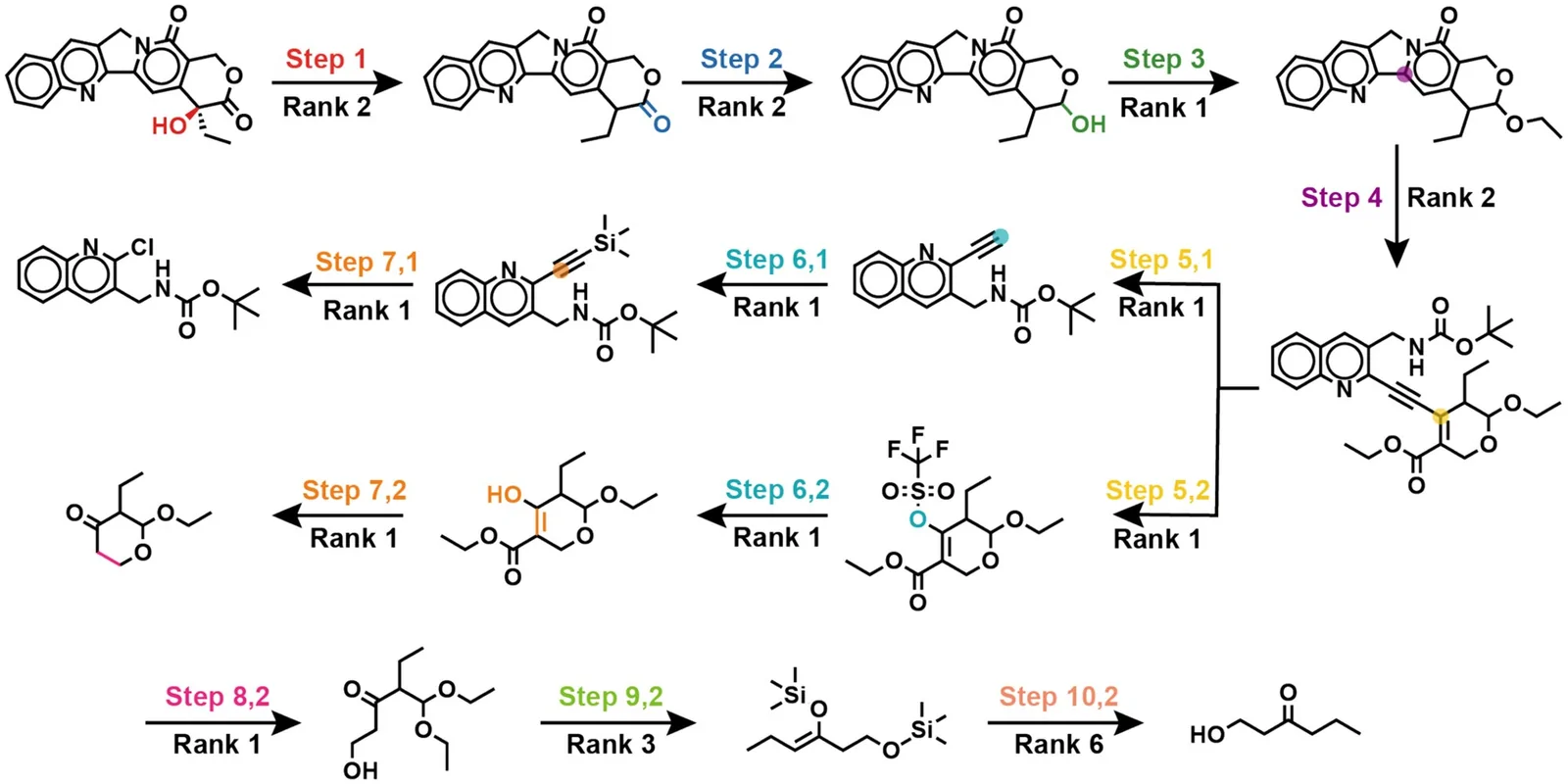
Camptothecin multistep retrosynthesis predictions by our method. The first precursor is 20-deoxycamptothecin; its C20(S) center is not a chirality error but is set later in the forward synthesis by an asymmetric *α*-hydroxylation (Tagami *et al*. 2000).

As shown in Fig. 4, ConfRetro was applied iteratively as a single-step predictor to deconstruct Camptothecin. The proposed pathway aligns closely with the literature synthesis (Li *et al.* 2016), confirming its feasibility. This robustness stems from our conformer-aware design, particularly Atom-align Fusion and Distance-weighted Attention, which enforce 3D structural and steric constraints during decoding.

**Figure 5:**
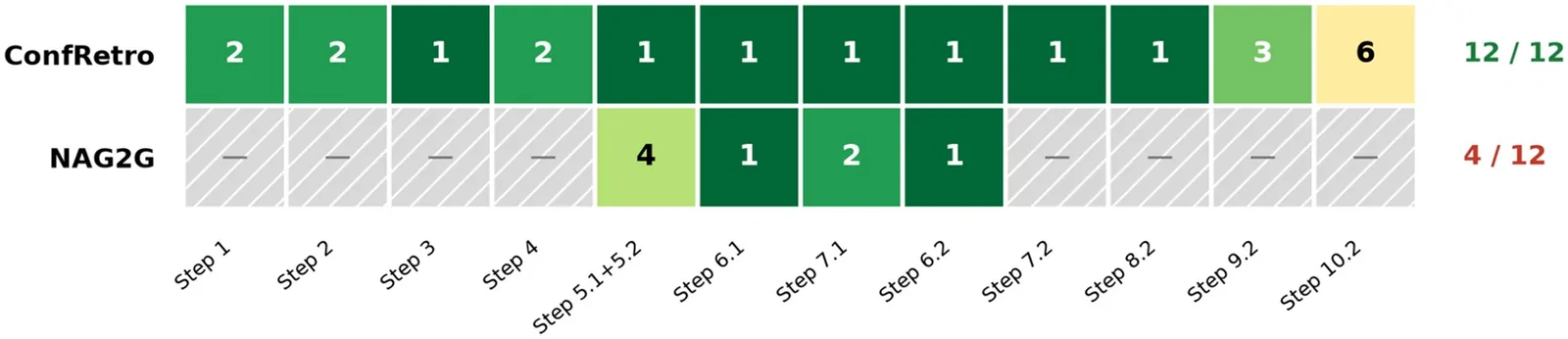
ConfRetro vs. NAG2G on the camptothecin route. Each cell gives the rank of the literature precursor at that step (lower is better; a dash denotes not recovered within the top-10 candidates).

To further assess whether the performance advantage stems from the proposed 3D design rather than from using 3D inputs per se, we compare ConfRetro against NAG2G, another 3D-aware template-free method, on the same Camptothecin route. As shown in Fig. 5, on this particular route ConfRetro recovers all 12 retrosynthetic steps within the top-6 predictions, whereas NAG2G recovers 4 steps, which are mostly the near-terminal building-block disconnections, and tends to miss several of the earlier ring-forming disconnections of the fused polycyclic core. While this is a single illustrative case rather than a quantitative benchmark, the observation is consistent with our single-step results and suggests that ConfRetro’s advantage may stem from how 3D information is integrated rather than merely from the availability of 3D inputs.

The results underscore the strength of our approach in handling intricate molecular architectures and its potential utility in accelerating the discovery and development of novel therapeutics. Additional synthesis routes for other representative molecules are provided in Supplementary Material H, available as supplementary data at *Bioinformatics* online.

## 6 Conclusion

We propose ConfRetro, an end-to-end Transformer that integrates 3D conformer information for retrosynthesis. With the proposed Atom-align Fusion module, we can adaptively integrate token and 3D position information while ensuring the alignment between them. Next, we propose a Distance-weighted Attention mechanism to guide and refine the redistribution of self-attention with spatial distances. Our model achieves a new state-of-the-art performance and template-free methods and is also highly competitive with template-based and semi-template methods. Furthermore, to validate the practicality of the model, we predicted synthetic routes for various candidate drug molecules, demonstrating its potential to revolutionize the prediction of complex chemical synthesis processes in future synthetic route design tasks. The results are provided in Supplementary Material H, available as supplementary data at *Bioinformatics* online.

## Supplementary Material

btag575_Supplementary_Data
