# Supplementary material for: ConfRetro: a 3D-aware template-free method for enhancing retrosynthesis via molecular conformer information
Source: Bioinformatics. 2026 Aug 5;42(8):btag575. doi: 10.1093/bioinformatics/btag575 (PMC13476070; doi:10.1093/bioinformatics/btag575)
Supplement: Supplementary file 1 [file btag575_supplementary_data.pdf]

A. Related Work

Template-based methods utilize reaction templates from pre-constructed database to capture the rules of chemical changes and match input products with the templates. RetroSim (Coley et al., 2017) is a similarity-based approach using molecular fingerprint similarity to rank candidate templates. GLN (Dai et al., 2019) employs the Conditional Graph Logic Network to learn when rules from reaction templates should be applied, implicitly considering chemical feasibility and strategy. Inspired by the chemical intuition that molecular changes primarily occur locally during reactions, LocalRetro (Chen and Jung, 2021) is locally encoded and refined to accommodate non-local reactions. Although these methods have strong interpretability, their reliance on pre-defined templates limits their coverage and scalability, rendering them insufficient for real-world scenarios.

Semi-template methods do not directly use templates but instead combine chemical rules with generative models. Existing methods typically follow a two-stage procedure: i) reaction center identification; ii) synthons completion. Reaction center identification predicts the reaction center of the product and breaks the corresponding bond into incomplete molecules, called synthons. Synthons completion uses generative models to complete synthons into reactants. RetroXpert (Yan et al., 2020) builds an Edge-enhanced Graph Attention Network (EGAT) that takes a molecule graph as input and predicts bond disconnection probabilities to obtain synthons. It then employs a seq2seq model to generate reactants SMILES. In contrast to SMILES format, G2Gs (Shi et al., 2020) completes synthons into reactants in graph format through node selection and edge labeling. GraphRetro (Somnath et al., 2020) expands synthons into complete reactants by attaching leaving groups. RetroPrime (Wang et al., 2021) introduces a two-stage procedure by adding additional labels to SMILES. In contrast, MEGAN (Sacha et al., 2021) ,MARS(Liu et al., 2024) and Graph2Edits (Zhong et al., 2023) reframe the generative process as a series of graph edits executed by `RDKit`.

Template-free methods transform retrosynthesis prediction (product→reactants) into a machine translation task (source language → target language) (Brown et al., 1990), adapting advanced NLP models like LSTM (Graves and Graves, 2012) and Transformer (Vaswani et al., 2017). Besides translation, SCROP (Zheng et al., 2019) builds a self-correction transformer to rectify the syntax errors. Augmented Transformer (Tetko et al., 2020) trains the model with data augmentation via SMILES permutation. To ensure the permutation invariance of SMILES, Graph2SMILES (Tu and Coley, 2022) substitutes the original sequence encoder with a graph encoder. GTA (Seo et al., 2021) masks the self-attention layer using the graph adjacency matrix. Retroformer (Wan et al., 2022) suggests a local attention head that enables information exchange between the local reactive region and the global reaction context. NAG2G (Yao et al., 2024) incorporates conformer as additional features but overlooks semantic alignment with SMILES and spatial receptive fields, failing to fully exploit the potential of 3D information.

B. USPTO-50K Dataset

B1. Performance across Each Reaction Class

In Table 3, we present the top-k accuracy (k=1, 3, 5, 10) of ConfRetro on the USPTO-50k dataset, where the reaction types are unknown during the training process. The results are categorized by the ground truth reaction types.

Our method seems to predict certain reaction types more accurately than other methods, especially those that are more concerned with 3D spatial structure, such as in Heterocycle formation, where the 3D structure of the molecule directly affects the stereoselectivity and yield of the cyclization reaction. It is challenging for other methods to deduce accurate results without 3D structural information.

Table 1. The top-k accuracy of each reaction class on USPTO-50k dataset, when trained with reaction class unknown.

Reaction Class	Reaction Fraction (%)	Top-k Accuracy (%)			
		1	3	5	10
Heteroatom alkylation and arylation	30.3	56.6	80.4	86.0	92.0
Acylation and related processes	23.8	68.5	89.9	94.3	97.7
Deprotections	16.5	54.5	78.5	85.9	91.9
C-C bond formation	11.3	41.6	63.1	73.9	83.4
Reductions	9.2	56.4	78.6	85.3	90.8
Functional group interconversion	3.7	39.4	56.4	64.6	76.1
Heterocycle formation	1.8	47.0	70.7	72.9	81.8
Oxidations	1.6	61.2	82.8	91.5	95.3
Protections	1.3	68.1	80.5	89.4	93.9
Functional group addition	0.5	80.2	85.2	89.6	98.4

B2. Performance on Different Conformer Generator

To explore the impact of different conformer generators on model performance, we conduct an ablation study. As shown in Table 5, in addition to the default `RDkit` generator, we evaluate the performance of the chemical simulation software `Schrödinger`, which is widely

regarded as capable of producing higher-quality conformations. The results show that the overall performance remains comparable. This observation suggests that our model exhibits strong robustness, as the quality of conformations has minimal impact on the final performance of ConfRetro.

Table 2. The performance with different conformer generator on USPTO-50k with reaction class unknown. All variants are evaluated with n_{aug}=1 for fair comparison. The Default (`RDKit`) model achieves 56.2/79.3/86.2/91.7 with n_{aug}=20 protocol (see main text Table 1).

**Conformer Generator**	**Top-k Accuracy (%)**			
	1	3	5	10
`Schrödinger` (Watts et al., 2010)	55.2	77.1	**83.8**	**90.1**
Default (`RDkit`)	**55.5**	**77.2**	83.4	89.1

B3. Performance on Different 3D Representation

This section explores the impact of 3D representations on model performance. Table 6 shows that as the 3D representation capacity increases (from top to bottom), the model's performance improves, indicating a positive correlation between them.

Table 3. The performance with different 3D representation networks on USPTO-50k with reaction class unknown. All variants are evaluated with n_{aug}=1 for fair comparison. The Default (ComENet) model achieves 56.2/79.3/86.2/91.7 with n_{aug}=20 protocol (see main text Table 1).

**3D Representation Network**	**Top-k Accuracy (%)**			
	1	3	5	10
SchNet (Schütt et al., 2018)	51.0	69.5	73.5	76.3
SphereNet (Coors et al., 2018)	53.1	77.1	83.1	87.8
Default (ComENet)	**55.5**	**77.2**	**83.4**	**89.1**

C. USPTO-FULL Datasets

We also use a more extensive collection, USPTO-FULL Dataset, which contains 1,013,118 atom-mapped reactions without any reaction class information. By experimenting with it, we can evaluate the proposed method's performance, generalization potential, and scalability, offering valuable insights into its practical application in retrosynthesis. Additionally, the dataset includes samples with **more intricate structures**, which allows for further validation of our model's capability to handle molecules with intricate spatial configurations.

C1. Preprocess

The USPTO-FULL raw data contains errors and other issues, so we implemented the following steps to clean and refine the dataset:

- Exclude reactions with invalid SMILES, including those with empty SMILES strings.
- Exclude reactions where the product contains atoms not present in the reactants.
- Exclude reactants if none of their atoms are found in the product.
- Exclude reactions where multiple atoms share the same atom map number.
- Exclude reactions where the product is composed of less than five atoms.

The final version shows an approximate 4% reduction compared to the raw data. The dataset is divided into training, validation, and test sets with sizes of 768K, 96K, and 96K, respectively.

D. Implementation Details

D1. 3D Distance Matrix Construction

In the Distance-weighted Attention mechanism, a 3D Distance Matrix needs to be constructed first. Algorithm 1 is the pseudo code for the construction of the 3D Distance Matrix $\mathbf{D}$ from a molecular canonical-SMILES S.

The algorithm begins by converting the SMILES into a molecular object M, followed by generating a conformer for the molecule and obtaining the coordinates of all atoms within it. Subsequently, an empty distance matrix is initialized. For each pair of atom tokens derived from tokenizing the SMILES, if both tokens represent atoms, the algorithm calculates the Euclidean distance between these two atoms and assigns this value to the corresponding position in the distance matrix; if they are not atom tokens, the algorithm sets the corresponding matrix element to 0. Finally, the algorithm returns the constructed 3D distance matrix $\mathbf{D}$.

Algorithm 1 3D Distance Matrix Construction

Input: molecule canonical SMILES S
Output: 3D Distance Matrix $\mathbf{D}$

Let $M = \text{MolFromSmiles}(S)$.
Generate conformer by $\text{MMFFOptimizeMolecule}(M)$.
Get coordinates of atoms $\mathbf{c} = \text{GetPositions}(M)$.
Initialize $\mathbf{D}$ as an empty array.
for $t_i, t_j \in \text{SmiTokenizer}(S)$ **do**
  if t_i, t_j are atom tokens **then**
    $\mathbf{D_{i,j}} = \text{norm}(\mathbf{c_i} - \mathbf{c_j})$
  else
    $\mathbf{D_{i,j}} = 0$
  end if
end for
return D

D2. Root-align SMILES Construction

Algorithm 2 is the pseudo code for the construction of the reactants Root-align SMILES S_R(+R) from a reactants canonical SMILES S.

First, the algorithm extracts the atom-mapped number from the product SMILES S_P, then iterates through the SMILES for each reactant, looking for the same atom-mapped number as in the S_P. Once a match is found, the algorithm uses the `RDkit` toolkit to generate a fixed-root SMILES centered on the corresponding atom and returns the constructed Root-align SMILES S_R(+R).

Algorithm 2 Root-align SMILES Construction

Input: atom-mapped product SMILES S_P and atom-mapped reactants SMILES S_R
Output: reactants R-SMILES S_R(+R)

Get atom-mapped number list $am(S_P)$ of S_P by Regular Expression Matching.
for $pNum \in am(S_P)$ **do**
  for $r_i \in \text{SmiTokenizer}(S_R)$ **do**
    if r_i is atom-mapped tokens and $am(r_i) == pNum$ **then**
      Make r_i as root-atom of S_R and get reactants R-SMILES S_R(+R)
      return S_R(+R)
    end if
  end for
end for
return S_R(+R)

D3. SMILES Alignment Map Construction

The ground truth SMILES Alignment Map SAM between the product SMILES S_P and the reactants SMILES S_R is computed as Algorithm 3.

The algorithm first initializes an empty SAM to store the correspondence between S_R and S_P. For each atom-mapped token in S_R, the algorithm looks for a corresponding token with the same atom-mapped number in S_P. Once this correspondence is found, the algorithm adds the pair of tokens to the SAM. This process will traverse forward and backward until there are no more matching tokens. Finally, the algorithm returns the constructed SMILES Alignment Map SAM.

Algorithm 3 SMILES Alignment Map Construction

Input: atom-mapped product SMILES S_P and atom-mapped reactants SMILES S_R
Output: SMILES Alignment Map SAM

Initialize SMILES Alignment Map SAM.
for do $r_i \in$ SmiTokenizer(S_R)
    if r_i is not visited and r_i is an atom-mapped token **then**
        Find the token $p_j \in$ SmiTokenizer(S_p) with the same atom-map number as r_i: $am(p_j) == am(r_i)$.
        while $r_i == p_j$ or $am(p_j) == am(r_i)$ **do**
            Add token alignment pair $\{r_i : p_j\}$ into SAM.
            $i = i + 1$ and $j = j + 1$.
        end while
    end if
end for
for $\{r_i : p_j\} \in SAM$ **do**
    $i = i - 1$ and $j = j - 1$.
    while $r_i == p_j$ and r_i is not an atom token **do**
        Add token alignment pair $\{r_i : p_j\}$ into SAM.
        $i = i - 1$ and $j = j - 1$
    end while
end for
return SAM

E. Hyperparameters Setting

Table 4. ConfRetro hyperparameters setup during training

**Hyperparameter**	**value**
Layers	6
Attention heads	8
Embedding dim	512
FFN hidden dim	2048
Gaussian kernel channels	512
FFN dropout	0.1
Attention dropout	0.1
Embedding dropout	0.1
Coefficient for KL-loss (α)	0.5
Activation function	ReLU
Vocabulary size	84
Batch size	16
Max training epoch	500
Beam Search Size	10
Warmup steps	8000
Weight decay	1e-3
Learning Rate Scheduler	Noam
Learning rate decay	0.001
Peak learning rate	23.8
Adams ϵ	0.5
Adams (β_1, β_2)	(0.9, 0.98)

F. Visualization

F1. Self-Attention Map

For better interpretability, we visualize the molecular self-attention maps and distance pair matrices as shown in Figure 1. It is evident that the sum of the spatial attention heads (Attention Heads 5, 6, 7, 8 in Figure 1) closely resembles the distance matrix, suggesting that spatial information has been integrated into the self-attention computation. However, when we check these heads independently, different patterns emerge. For example, the normal attention head weights are asymmetric, focusing more on self-token interactions,

whereas the distance matrix tends to interact with other tokens. Additionally, long-range interactions can be captured through spatial attention (such as in Attention Heads 7 and 8).

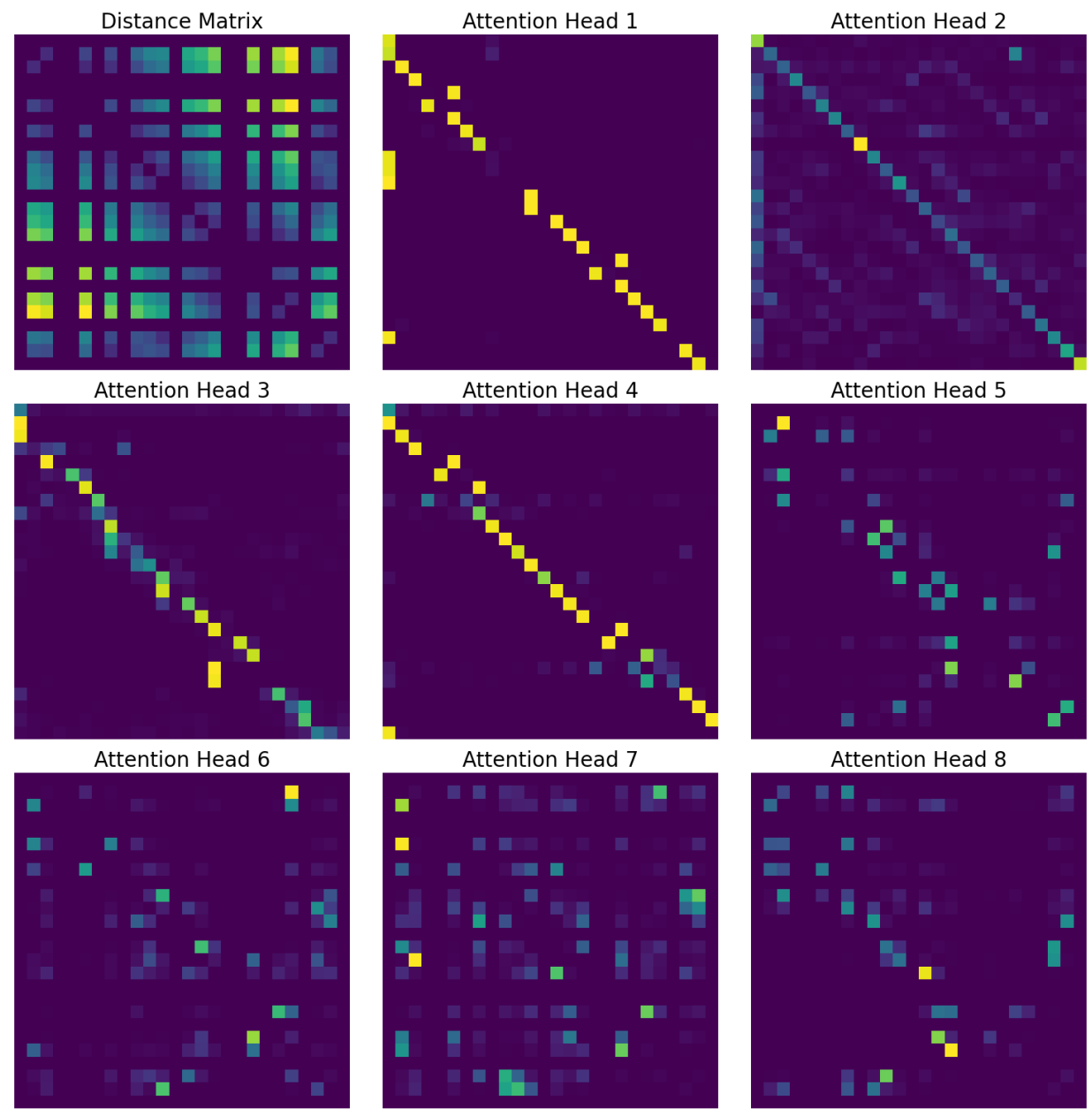

Fig. 1: Visualization on self-attention map of multi heads independently.

F2. Cross-Attention Map

SMILES Alignment between product and reactants and Cross Attention Score on first head, respectively. The left most figure is the ground truth alignment matrix. It is easy to find that cross-attention captures the corresponding information of the SMILES Alignment Map very well and assists the model generation process in the Decoder stage, which also promotes the final excellent performance of our model.

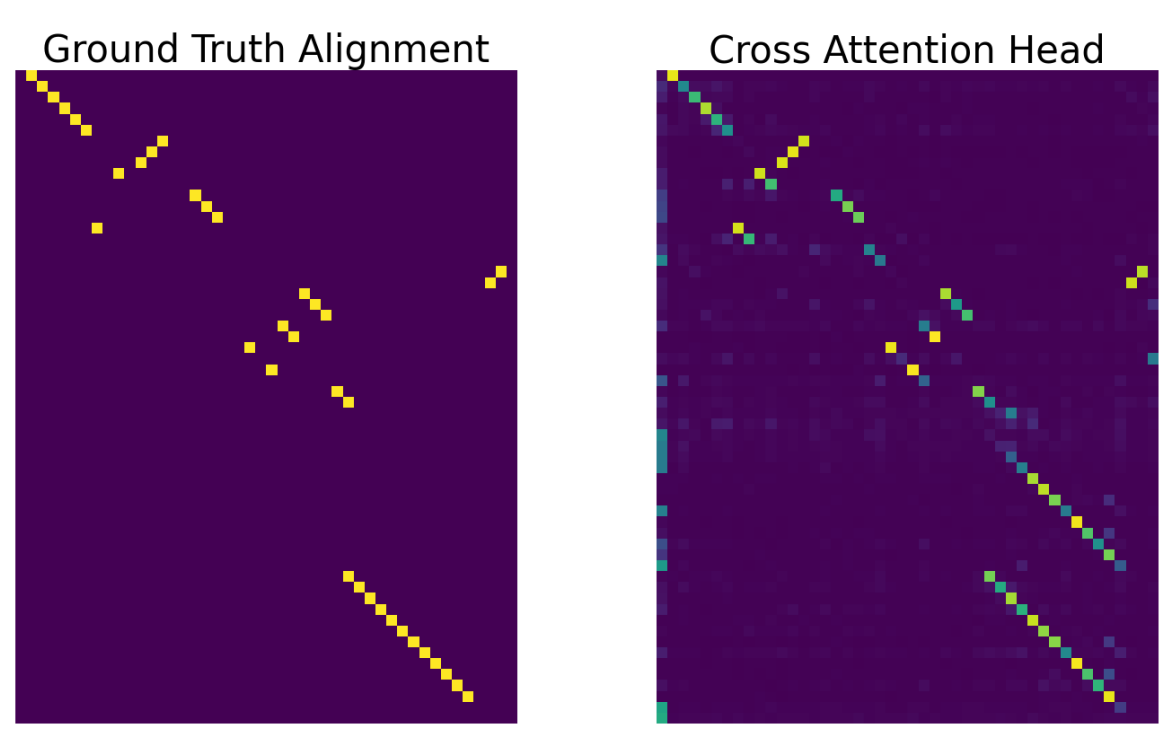

Fig. 2: Visualization on cross-attention map on SMILES Alignment.

G. Case Study

Here we show **statistic analysis** and **more illustrations** in Case Study on **Intricate Molecules**.

Table 5. Performance comparison of ConfRetro, NAG2G, and Vanilla Transformer across four types of molecular structures on USPTO-50K (reaction class unknown). Best per column in **bold**.

Type	Model	Top-k Accuracy (%) 1	3	5	10	Top-k Validity (%) 1	3	5	10
**Polychiral**	Trans.	38.5	59.0	67.5	73.9	97.3	97.3	96.2	92.6
	NAG2G	41.9	63.9	71.2	77.6	**99.6**	**98.3**	96.5	91.5
	**ConfRetro**	**45.7**	**66.2**	**75.7**	**83.7**	98.6	98.1	**97.4**	**96.2**
**Heteroaromatic**	Trans.	51.2	71.9	79.0	84.7	98.2	97.6	96.4	91.1
	NAG2G	56.2	77.5	83.9	90.2	**99.7**	98.7	97.2	92.9
	**ConfRetro**	**56.8**	**79.3**	**86.1**	**91.8**	99.3	**98.9**	**98.7**	**96.1**
**Fused / Bridged Rings**	Trans.	50.8	71.1	77.8	83.3	99.2	98.1	97.1	91.6
	NAG2G	55.6	76.1	82.5	89.3	**99.5**	**98.5**	96.8	92.3
	**ConfRetro**	**56.8**	**78.0**	**85.1**	**91.1**	99.4	98.4	**97.9**	**95.8**
**Complex**	Trans.	49.5	70.3	77.5	83.2	98.3	97.6	96.3	92.0
	NAG2G	54.7	75.4	81.6	88.5	**99.6**	98.6	96.9	92.3
	**ConfRetro**	**55.7**	**77.4**	**84.5**	**90.4**	99.4	**98.8**	**98.1**	**95.5**

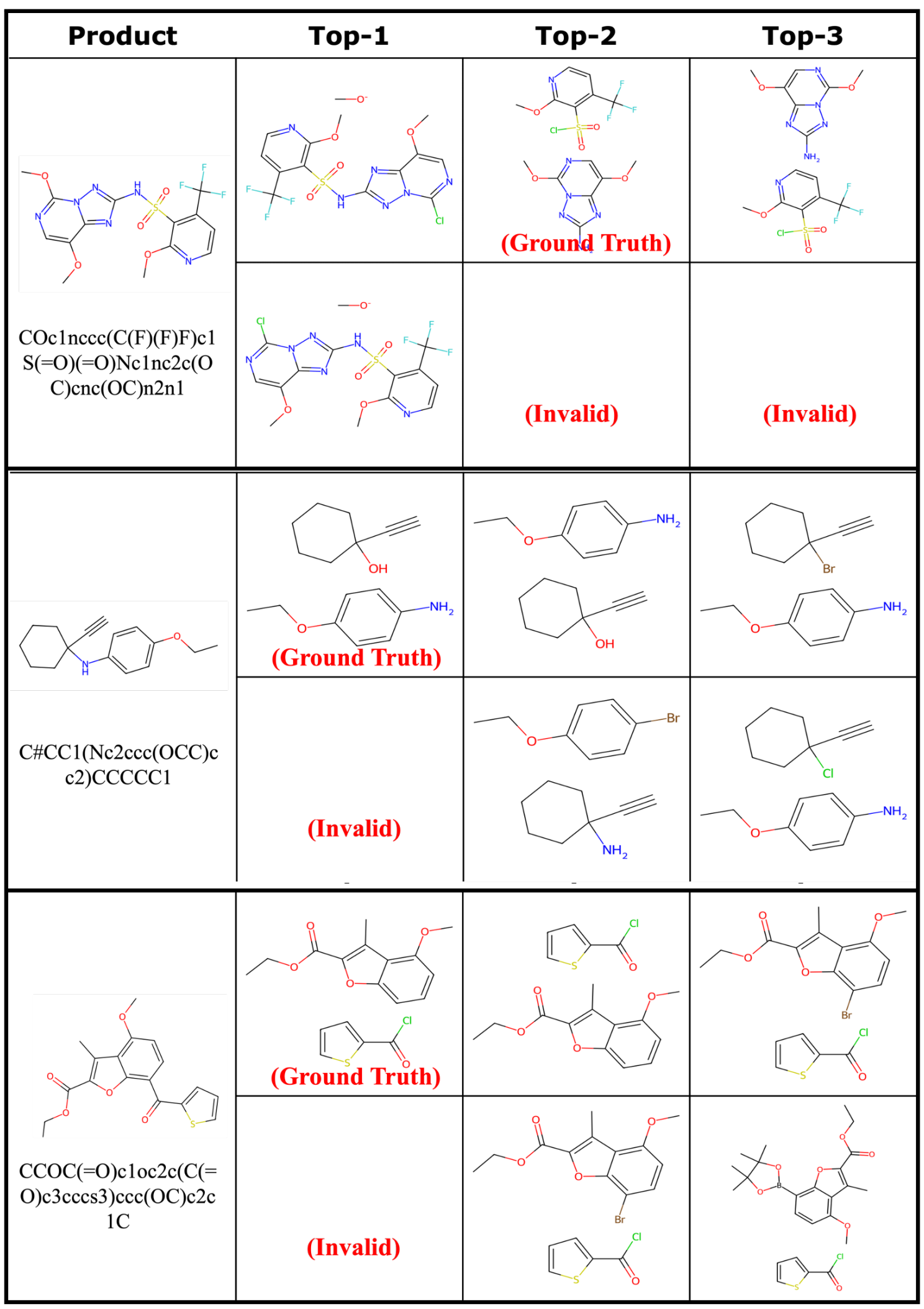

Fig. 3: More Case. Each subfigure shows predictions of different methods: **ConfRetro (Top)**, **Transformer (Bottom)**.

H. Synthesis route

By applying our product-reactant variant recursively, we verify our method with several multistep retrosynthesis examples reported in the literature, including febuxostat (Cao et al., 2016), salmeterol (Guo et al., 2011), an allosteric activator for GPX_4 (Lin et al., 2020), a 5-HT_6 receptor ligand (Nirogi et al., 2015), Nirmatrelvir (Hammond et al., 2022) and Osimertinib (Finlay and others., 2014). As shown in Fig. 4 , our method successfully predicts the complete synthetic pathway for these examples.

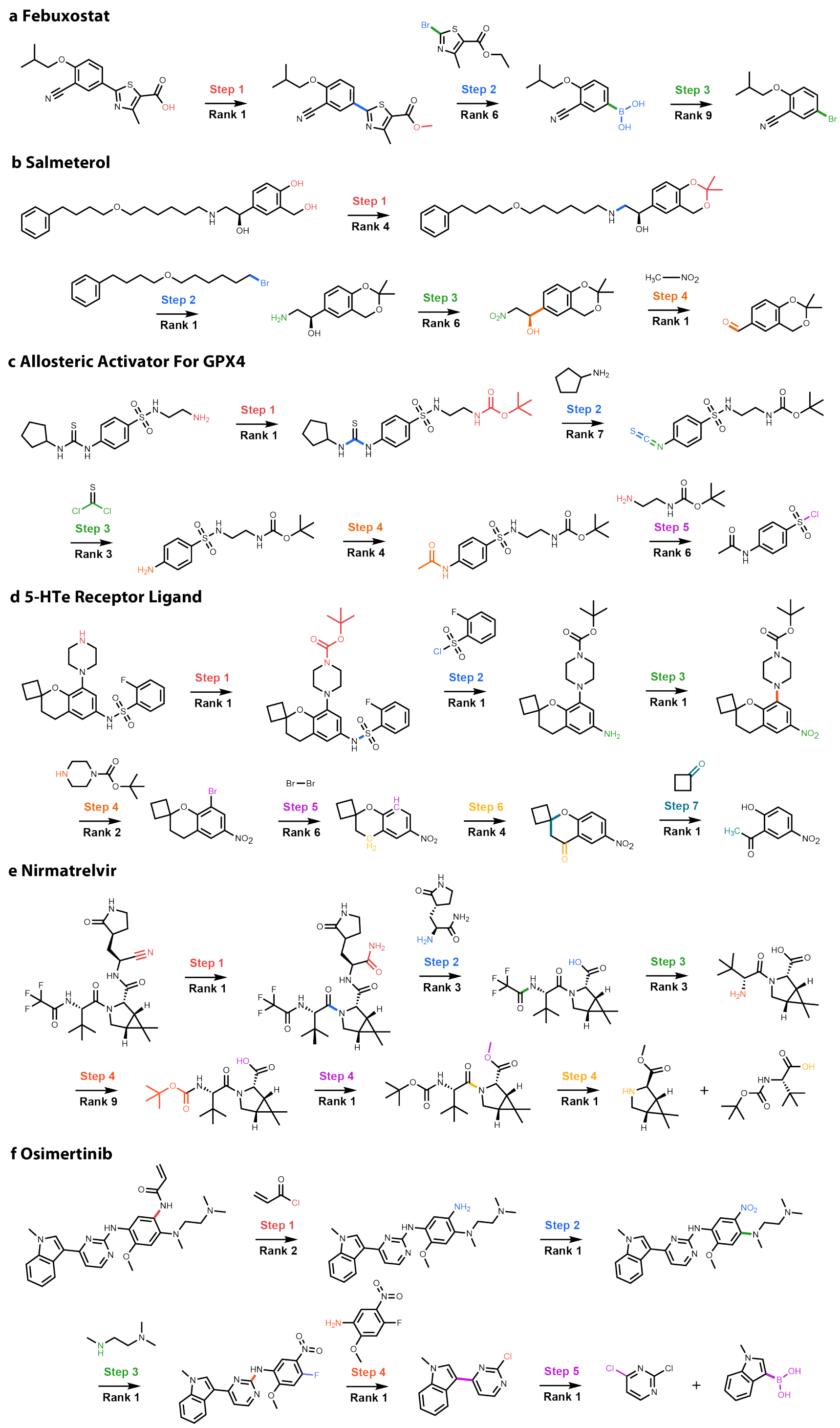

Fig. 4: Multistep retrosynthesis predictions by our method. (a) Febuxostat. (b) Salmeterol. (c) Allosteric Activator for GPX4. (d) 5-HTe Receptor Ligand.

I. Robustness and Stability Analysis

I1. Multi-Seed Training Stability

To assess the reproducibility of reported results, we trained ConfRetro with five independent random seeds $\{10, 1, 42, 123, 2024\}$ under the same protocol (USPTO-50K, reaction class unknown, n_{aug}=20, beam 10). Table 6 reports per-seed results together with the mean and standard deviation.

Table 6. Top-k accuracy across five random seeds (USPTO-50K, reaction class unknown).

Seed	Top-1	Top-3	Top-5	Top-10
10	56.2	79.3	86.2	91.7
1	56.5	79.8	86.7	92.0
42	55.9	78.8	85.7	91.5
123	56.3	79.5	86.4	91.7
2024	56.1	79.1	86.0	91.6
**Mean ± Std**	**56.2±0.2**	**79.3±0.4**	**86.2±0.4**	**91.7±0.2**

The Top-1 standard deviation is ±0.2%, confirming that reported results are stable across training runs.

I2. Conformer-Seed Robustness

To verify that performance is insensitive to the specific RDKit conformer used at test time, we evaluated a trained ConfRetro model on five different RDKit embedding seeds. Table 7 reports per-seed results together with the mean and standard deviation. The Top-1 standard deviation is ±0.1%, demonstrating that ConfRetro's performance is essentially invariant to which conformer is used. A five-conformer reciprocal-rank fusion ensemble achieves 56.3/79.4/86.3/91.8, on par with a single conformer, confirming that a cheap single deterministic conformer suffices.

Table 7. Top-k accuracy across five RDKit conformer-embedding seeds (USPTO-50K, reaction class unknown). Seed 10 is the default conformer used throughout the paper.

Seed	Top-1	Top-3	Top-5	Top-10
10	56.2	79.3	86.2	91.7
7	56.1	79.2	86.1	91.4
23	56.3	79.4	86.3	91.9
88	56.2	79.3	86.2	91.7
256	56.2	79.3	86.2	91.8
**Mean ± Std**	**56.2±0.1**	**79.3±0.1**	**86.2±0.1**	**91.7±0.2**

I3. Stereochemistry-Stratified Evaluation

To verify that ConfRetro's gains are not confounded by stereochemical annotation quality, we stratified the USPTO-50K test reactions by the stereochemistry of the product into three groups: *defined* (the product has at least one stereocentre whose configuration is explicitly assigned in the SMILES, e.g. `@`/`@@` or E/Z; n=870, 17.4%); *unspecified* (the product contains at least one stereocentre, but its configuration is left unassigned in the SMILES, i.e. a potential chiral atom without an `@`/`@@` label; n=940, 18.8%); and *none* (the product has no stereocentre at all; n=3,180, 63.7%). The *defined* and *unspecified* groups thus differ only in whether the existing stereocentres carry an explicit configuration. In each stratum we compare the standard ConfRetro, which uses the real RDKit conformer, against a *random-conformer* control: an otherwise identical model whose 3D atomic coordinates are replaced by random noise (the same control as "w/ random conformer" in main-text Table 5), so that the gap isolates the contribution of genuine 3D geometry. Table 8 reports the results per stratum.

ConfRetro outperforms the random-conformer control across all strata, with the largest Top-1 gain on the *unspecified* stratum (+3.5), showing that the model benefits from 3D geometry even when stereo annotations are absent or ambiguous.

I4. Checkpoint-Count Ablation

Table 9 reports the effect of the number of averaged checkpoints. Top-1 accuracy rises from 55.4 (3 checkpoints) to 56.2 (7 checkpoints) then plateaus; we therefore selected k=7 as the best and most efficient choice.

Table 8. Stereochemistry-stratified Top-k accuracy: ConfRetro (real conformer) vs. the random-conformer control (3D coordinates replaced by random noise).

Stratum	Model	Top-1	Top-3	Top-5	Top-10
All	ConfRetro	56.2	79.3	86.2	91.7
	Random	53.4	77.7	84.8	90.7
Defined	ConfRetro	51.7	71.0	80.0	86.8
	Random	49.4	69.7	76.7	84.0
Unspecified	ConfRetro	52.0	76.5	83.8	89.4
	Random	48.5	73.4	81.6	88.2
None	ConfRetro	58.6	82.5	88.7	93.8
	Random	56.0	81.2	88.0	93.3

Table 9. Effect of checkpoint averaging count on USPTO-50K (reaction class unknown).

Checkpoints	Top-1	Top-3	Top-5	Top-10
3	55.4	78.5	85.4	91.3
5	55.9	78.9	85.8	91.5
**7 (selected)**	**56.2**	79.3	86.2	91.7
9	56.1	79.2	86.1	91.6

I5. Statistical Significance Tests

To confirm that the accuracy gaps are real rather than test-set noise, we run two analyses on the 4,990 USPTO-50K test reactions (reaction class unknown). *(i) Bootstrap confidence intervals (CIs).* We resample the test reactions with replacement B=10,000 times and take the 2.5th and 97.5th percentiles of each metric as its 95% CI (Table 10); a narrow CI means a stable estimate, and two CIs that barely overlap indicate a real gap. *(ii) McNemar tests.* Since all models are tested on the same reactions, we compare them in a paired manner using the exact two-sided McNemar test, which looks only at the reactions solved by exactly one of the two models at Top-1 and asks whether such disagreements are balanced; a small p-value means they are too one-sided to be due to chance (Table 11). The *Diff* column gives the Top-1 accuracy difference (A−B) with its bootstrap 95% CI.

Table 10. Bootstrap 95% confidence intervals (percentile, B=10,000) on USPTO-50K.

Model	Top-1	Top-3	Top-5	Top-10
ConfRetro	56.2 [54.8, 57.5]	79.3 [78.2, 80.4]	86.2 [85.2, 87.2]	91.7 [90.9, 92.5]
w/o bond-type	56.0 [54.5, 57.4]	79.2 [78.1, 80.4]	86.5 [85.5, 87.4]	91.8 [91.0, 92.6]
w/ random conformer	53.4 [52.1, 54.8]	77.7 [76.5, 78.9]	84.8 [83.8, 85.8]	90.7 [89.9, 91.5]
w/o 3D distance	53.0 [51.5, 54.3]	77.4 [76.3, 78.6]	84.5 [83.5, 85.5]	90.5 [89.6, 91.3]
Retroformer	53.6 [52.3, 55.0]	73.7 [72.4, 74.9]	80.2 [79.0, 81.3]	86.1 [85.1, 87.0]

Table 11. Paired significance: difference in accuracy and McNemar p-value.

A	B	Diff (A−B)	McNemar p
ConfRetro	w/ random conformer	+2.71 [+1.72, +3.67]	9.0×10^{-8}
ConfRetro	w/o 3D distance	+3.19 [+2.18, +4.19]	4.3×10^{-10}
w/o bond-type	w/o 3D distance	+3.01 [+2.04, +3.97]	1.6×10^{-9}
ConfRetro	Retroformer (Top-1)	+2.55	1.9×10^{-5}
ConfRetro	Retroformer (Top-3)	+4.73	5.2×10^{-20}
ConfRetro	Retroformer (Top-5)	+4.99	7.0×10^{-27}
ConfRetro	Retroformer (Top-10)	+5.03	2.4×10^{-37}

All geometry-related improvements are statistically significant at $p < 10^{-6}$.

References

P. F. Brown, J. Cocke, S. A. Della Pietra, V. J. Della Pietra, F. Jelinek, J. Lafferty, R. L. Mercer, and P. S. Roossin. A statistical approach to machine translation. *Computational linguistics*, 16(2):79–85, 1990.

Q. M. Cao, X. L. Ma, J. M. Xiong, P. Guo, and J. P. Chao. The preparation of febuxostat by suzuki reaction. *Chin. J. New Drugs*, 2016.

S. Chen and Y. Jung. Deep retrosynthetic reaction prediction using local reactivity and global attention. *JACS Au*, 1(10):1612–1620, 2021.

C. W. Coley, L. Rogers, W. H. Green, and K. F. Jensen. Computer-assisted retrosynthesis based on molecular similarity. *ACS central science*, 3(12):1237–1245, 2017.

B. Coors et al. Spherenet: Learning spherical representations for detection and classification in omnidirectional images. In *Proceedings of the European conference on computer vision (ECCV)*, pages 518–533, 2018.

H. Dai, C. Li, C. Coley, B. Dai, and L. Song. Retrosynthesis prediction with conditional graph logic network. *Advances in Neural Information Processing Systems*, 32, 2019.

M. R. V. Finlay and others. Discovery of a potent and selective egfr inhibitor (azd9291) of both sensitizing and t790m resistance mutations that spares the wild type form of the receptor. *Journal of Medicinal Chemistry*, 57:8249–8267, 2014. doi: 10.1021/jm500973a.

A. Graves and A. Graves. Long short-term memory. *Supervised sequence labelling with recurrent neural networks*, pages 37–45, 2012.

Z.-L. Guo, Y.-Q. Deng, S. Zhong, and G. Lu. Enantioselective synthesis of (r)-salmeterol employing an asymmetric henry reaction as the key step. *Tetrahedron: Asymmetry*, 22(13):1395–1399, 2011.

J. Hammond et al. Oral nirmatrelvir for high-risk, nonhospitalized adults with covid-19. *New England Journal of Medicine*, 386(15): 1397–1408, 2022.

K. Lin, Y. Xu, J. Pei, and L. Lai. Automatic retrosynthetic route planning using template-free models. *Chem. Sci.*, 11(12):3355–3364, 2020.

J. Liu, C. Yan, Y. Yu, C. Lu, J. Huang, L. Ou-Yang, and P. Zhao. Mars: a motif-based autoregressive model for retrosynthesis prediction. *Bioinformatics*, 40(3):btae115, 2024.

R. V. Nirogi, R. Badange, V. Reballi, and M. Khagga. Design, synthesis and biological evaluation of novel benzopyran sulfonamide derivatives as 5-ht6 receptor ligands. *Asian J. Chem.*, 2015.

M. Sacha, M. Błaz, P. Byrski, P. Dabrowski-Tumanski, M. Chrominski, R. Loska, P. Włodarczyk-Pruszynski, and S. Jastrzebski. Molecule edit graph attention network: modeling chemical reactions as sequences of graph edits. *Journal of Chemical Information and Modeling*, 61(7):3273–3284, 2021.

K. T. Schütt, H. E. Sauceda, P.-J. Kindermans, A. Tkatchenko, and K.-R. Müller. Schnet–a deep learning architecture for molecules and materials. *The Journal of Chemical Physics*, 148(24), 2018.

S.-W. Seo, Y. Y. Song, J. Y. Yang, S. Bae, H. Lee, J. Shin, S. J. Hwang, and E. Yang. Gta: Graph truncated attention for retrosynthesis. In *Proceedings of the AAAI Conference on Artificial Intelligence*, volume 35, pages 531–539, 2021.

C. Shi, M. Xu, H. Guo, M. Zhang, and J. Tang. A graph to graphs framework for retrosynthesis prediction. In *International conference on machine learning*, pages 8818–8827. PMLR, 2020.

V. R. Somnath, C. Bunne, C. W. Coley, A. Krause, and R. Barzilay. Learning graph models for template-free retrosynthesis. *arXiv preprint arXiv:2006.07038*, 2020.

I. V. Tetko, P. Karpov, R. Van Deursen, and G. Godin. State-of-the-art augmented nlp transformer models for direct and single-step retrosynthesis. *Nature communications*, 11(1):5575, 2020.

Z. Tu and C. W. Coley. Permutation invariant graph-to-sequence model for template-free retrosynthesis and reaction prediction. *Journal of chemical information and modeling*, 62(15):3503–3513, 2022.

A. Vaswani, N. Shazeer, N. Parmar, J. Uszkoreit, L. Jones, A. N. Gomez, Ł. Kaiser, and I. Polosukhin. Attention is all you need. *Advances in neural information processing systems*, 30, 2017.

Y. Wan, C.-Y. Hsieh, B. Liao, and S. Zhang. Retroformer: Pushing the limits of end-to-end retrosynthesis transformer. In *International Conference on Machine Learning*, pages 22475–22490. PMLR, 2022.

X. Wang, Y. Li, J. Qiu, G. Chen, H. Liu, B. Liao, C.-Y. Hsieh, and X. Yao. Retroprime: A diverse, plausible and transformer-based method for single-step retrosynthesis predictions. *Chemical Engineering Journal*, 420:129845, 2021.

K. S. Watts, P. Dalal, R. B. Murphy, W. Sherman, R. A. Friesner, and J. C. Shelley. Confgen: a conformational search method for efficient generation of bioactive conformers. *Journal of chemical information and modeling*, 50(4):534–546, 2010.

C. Yan, Q. Ding, P. Zhao, S. Zheng, J. Yang, Y. Yu, and J. Huang. Retroxpert: Decompose retrosynthesis prediction like a chemist. *Advances in Neural Information Processing Systems*, 33:11248–11258, 2020.

L. Yao, W. Guo, Z. Wang, S. Xiang, W. Liu, and G. Ke. Node-aligned graph-to-graph: Elevating template-free deep learning approaches in single-step retrosynthesis. *JACS Au*, 4(3):992–1003, 2024. doi: 10.1021/jacsau.3c00737.

S. Zheng, J. Rao, Z. Zhang, J. Xu, and Y. Yang. Predicting retrosynthetic reactions using self-corrected transformer neural networks. *Journal of chemical information and modeling*, 60(1):47–55, 2019.

W. Zhong, Z. Yang, and C. Y.-C. Chen. Retrosynthesis prediction using an end-to-end graph generative architecture for molecular graph editing. *Nature Communications*, 14(1):3009, 2023.
